# Evolution of quality of life in patients with breast cancer during the first year of follow-up in Morocco

**DOI:** 10.1186/s12885-018-4008-3

**Published:** 2018-02-06

**Authors:** B. M. Traore, S. El Fakir, H. Charaka, N. Benaicha, A. Najdi, A. Zidouh, M. Bennani, H. Errihani, N. Mellass, A. Benider, R. Bekkali, C. Nejjari

**Affiliations:** 1 Department of epidemiology and public health, Faculty of Medicine, University Sidi Mohammed Ben Abdellah, Fez, Morocco; 2 Fondation Lalla Salma Prevention and Treatment of Cancers, Rabat, Morocco; 3 Oncology Center Ibn Rochd, Casablanca, Morocco; 4 National Institute of Oncology, Rabat, Morocco; 5 Department ofOncology, University Hospital Center Hassan II Fez, Fez, Morocco

**Keywords:** Questionnaire, Quality of life, Breast cancer

## Abstract

**Background:**

Quality of life has an important place in the future of patients with breast cancer. The objective of this study is to assess the evolution of the patient’s quality of life with breast cancer in Morocco after a year of follow-up.

**Methods:**

This study involved the patients with breast cancer with all types of treatment as determined by their physicians. Patient’s quality of life was assessed with the Moroccan Arabic version of QLQ- EORTC QLQ C30 and EORTC-BR23 questionnaires. Data were analyzed using SPSS Version 20 software.

**Results:**

Regarding EOTRC questionnaires QLQ C-30, there was a significant improvement in global health status and all scales of the functional dimension except the social functional where there was a trend towards improvement and the financial impact of the disease where the situation has deteriorated. Quality of life was improved for most symptom-sized scales dimension of EORTC QLQ- C30 with the exception of diarrhea where it was observed degradation. Most of the EORTC QLQ-scales BR23 questionnaires showed a favorable trend in the quality of life except those of sexual functioning, sexual enjoyment, hair loss and the side effects of systemic therapy.

**Conclusion:**

The quality of life of the patient is significantly improved after 1 year of follow up. Quality of life instruments can be useful in the early identification of patients whose score low on functional scales and symptoms.

## Background

Breast cancer (BC) is the most common malignancy in women worldwide. Currently, breast cancer incidence in Europe is 94.3 per 100,000, with a mortality of 26 per 100,000 [1]. BC accounts for one third of cancers diagnosed in women in United States and is the second leading cause of cancer death worldwide [2].

During the period 2002-2007, mortality rates from BC decreased 6.9% in the European Union and 6.3% in Lithuania. About 70-80% of patients with breast cancer are still alive, and quality of life (QoL) has an important place in women’s well-being [3].

QoL is the appropriate one of the main determinants of treatment success in modern oncology [4].QoL related to health is now considered as an important parameter in clinical cancer trials. It has been shown that quality of life assessment in cancer patients to help improve treatment and may even be one of prognostic factors [5, 6]. To assess quality of life, multiple scales can be used. In oncology, the questionnaire on the quality of life of the European Organization for Research and Treatment of Cancer (EORTC QLQ-C30) and specific module of the breast QLQ-BR23 [7] are the most useful probably because they are reliable, simple, available and easy to answer and validated in several European languages.

Quality of life measurement instruments have been widely used in many global tests. Studies indicate that the scales of quality of life provide prognostic information in addition to sociodemographic and clinical measures, and also can help predict survival in patients with breast cancer [8].

In Morocco, the introduction of the concept of quality of life is recent. Moroccan Arabic dialect versions EORTC QLQ-questionnaires C30, QLQ-BR23 have been validated and can be used to assess changes in the quality of life of patients with breast cancer [9].

The objective of this study is to evaluate the evolution of quality of life of Moroccan patient with breast cancer during the first year of follow-up.

## Methods

### Population study and data collection

This is a multicenter, prospective observational study on quality of life of breast cancer patients. It has been carried out in the main cancer centers in the country (National Oncology Institute in Rabat, Ibn Rochd Hospital in Casablanca, the hospital’s oncology center Mohamed VI in Marrakech, oncology center the Hassan II Hospital in Fez, Oujda cancer center, cancer center in Agadir).

Patients were recruited during the period of 2009-2011. They were followed for 1 year to assess changes in their quality of life. This follow-up was done at 1 month and 12 months of their inclusion in the study. They were included in the study with any type of treatment, as determined by their physicians. The survey was conducted by trained physicians using the assessment of quality of life questionnaires (EORTC QLQ-C30 and EORTC QLQ-BR23).

### Ethics approval and consent to participate

Ethical approval was obtained with the ethics committee of the hospital Hassan II of Fez, Morocco. All participants were informed of the study conditions and gave written informed consent.

### Measure

Moroccan Arabic version of the EORTC QLQ-C30 and its supplementary breast cancer questionnaire EORTC QLQ-BR23 have been validated to assess quality of life in patients with cancer and particularly in patients suffering from breast cancer in our study.

EORTC QLQ-C30 includes 30 items divided between a functional dimension and dimension symptoms. The functional dimension is composed of physical scales, emotional, cognitive, social and professional activity. The symptom dimension consists of fatigue scales, pain and nausea / vomiting. In addition we have a global health scale, five scales simple symptoms (dyspnea, insomnia, loss of appetite, constipation and diarrhea) and a scale assessing perceived financial impact of the disease.

EORTC QLQ-BR23, breast cancer specific questionnaire consists of 23 items divided between a functional dimension scales including: body image, sexual functioning, sexual enjoyment and future prospects and a symptom dimension consists of systemic therapy scales, side effects, breast symptoms, hands and hair loss symptoms.

According to the guidelines of the EORTC, scores on the items were converted to a scale of 0 to 100.A high score for a functional scale represents a healthy level of functioning, a high score for the overall health status represents a high quality of life, but a high score on a scale of symptoms post represents a high level of symptomatology [9].

### Statistical analysis

Statistical analysis initially consisted in a description of our population study. Categorical variables were expressed in proportion while Quantitative variables were described by the mean and standard deviations.

For the assessment of the quality of life, the Student’s test for the comparison of means paired data was used to search for the possible existence of differences in life of quality between the different parameters in the first and twelfth month for each scale EORTC-C30 and EORTC-BR23. Data were analyzed using SPSS Version 20.0 software.

## Results

A total of 1463 women were included in the study. The mean age was 50.51 ± 10.92 years with extremes of 21 and 98 years. Less than 50 years age group was the most affected with 54.5%. Women for the most part lived in urban region (72.9%), were illiterate for the majority (61.7%) and housewives in 75.6%. Most women had low socioeconomic status (66.8%), were married in 70.1%. Only 26.9% of women had a social security. The disease was in stage 2 for 41.4% of women (Table 1).

**Table 1:** sociodemographic characteristics of the study population

Characteristics	Percentage (%)
Age group (years) *N* = 1463	
< 50	54,6
50-59	25,6
60-69	13,9
≥ 70	5,9
Place of residence (*N* = 1330)	
Urban	72,9
Rural	27,1
Education (*N* = 1463)	
illiterate	61,7
literate	38,3
Marital status (*N* = 1463)	
Single	13,4
Married	70,1
Divorced	5,9
widowed	10,6
Professional status (*N* = 1463)	
Housewives	75,6
Unemployed	11
In professional activity	13,4
Social level (*N* = 1463)	
Low	66,8
Mean	31,5
high	1,7
Social Security (*N* = 1463)	
No	73,1
Yes	26,9
Stage disease (*N* = 1411)	
Stage 1	14,88
Stage 2	42,95
Stage 3	28,85
Stage 4	13,32

Changes in QoL were assessed at the first and twelfth months. Different parameters of EORTC QLQ-C30 and EORTC QLQ-BR23 questionnaires were evaluated. Regarding EORTC QLQ-C30, Global health status improved during follow-up (66.67 vs 76.02, *p* < 0.001). Almost all of the functional dimension scores showed significant improvement between measurements at 1 month and 12 months, except social activity where there’s a trend of improvement. (87.85 vs. 88, 53, *p* = 0.473).

Significant improvements were observed for symptoms dimension on fatigue scales, pain, insomnia and anorexia. However regarding dyspnea, nausea / vomiting and constipation, there was a tendency for improvement. QoL has worsened for the diarrhea scale (4.41 vs. 5.33, *p* = 0.002). Financial conditions also deteriorated (66.67 vs 33.33, *p* = 0.001) (Table 2).

**Table 2:** Scores scales of ERTC QLQ-C30 questionnaire

		Means ± Standard Deviation		
	Frequency	1st month	12th month	*P* value
Functional scales				
Global Health Status	691	66,67 ± 17,76	76,02 ± 17,74	< 0,001
Physical functioning	695	80,88 ± 19,31	84,69 ± 18,64	< 0,001
Role functioning	694	75,65 ± 27,58	83,69 ± 23,28	< 0,001
Emotional Functioning	692	63,01 ± 28,59	73,06 ± 25,10	< 0,001
Cognitive functioning	690	84,83 ± 22,77	88,36 ± 20,47	< 0,001
Social functioning	690	87,85 ± 21,76	88,53 ± 19,40	0,473
Symptom scales				
Fatigue	689	26,83 ± 22,69	20,86 ± 22,47	< 0,001
Nausea and Vomiting	692	8,04 ± 18,29	6,79 ± 17,09	0,104
Pains	695	21,85 ± 25,47	14,89 ± 21,89	< 0,001
Dyspnea	690	13,04 ± 23,01	11,93 ± 22,59	0,288
Insomnia	686	21,10 ± 27,62	13,98 ± 23,83	< 0,001
Appetite loss	687	21,70 ± 27,63	10,90 ± 21,50	< 0,001
Constipation	690	9,08 ± 20,97	7,25 ± 19,56	0,058
Diarrhea	688	4,41 ± 11,88	5,33 ± 15,39	0,002
Financial difficulties	681	66,67 ± 37,62	33,33 ± 37,29	< 0,001

For EORTC QLQ-of BR23, body image and future prospects have clearly improved during the study period. Sexual functioning which had a high score in the first months slightly worsened at 12 months (76.69 vs 69.84, *p* < 0.001); It is also the same for sexual enjoyment (55.60 vs 53.14 p < 0.001). For the symptoms dimension, significant improvements were observed for symptoms of “breast symptoms” and “arms”, while we noted depreciation of the quality of life for scales of “side effects” and “hair loss” (Table 3).

**Table 3:** Scores scales of ERTC QLQ-BR23 questionnaire

		Means ± Standard Deviation		
	Frequency	1st month	12th month	*P* value
Functional scales				
Body image	669	81,88 ± 23,32	85,52 ± 20,50	< 0,001
Sexual functional	462	76,69 ± 23,68	69,84 ± 22,19	< 0,001
Sexual enjoyment	244	55,60 ± 29,64	53,14 ± 30,30	< 0,001
Future Perspective	662	39,78 ± 37,23	46,68 ± 38,13	< 0,001
Symptom scales				
systemic therapy side effects	685	16,86 ± 17,04	17,16 ± 17,77	< 0,001
Breast Symptoms	650	18,71 ± 19,97	15,04 ± 19,02	< 0,001
Arm Symptoms	661	22,52 ± 21,16	18,92 ± 19,39	< 0,001
Upset by hair loss	151	20,97 ± 27,65	22,96 ± 26,72	0,003

## Discussion

This study allowed to analyze the evolution of the quality of life in patients with breast cancer. All patients were included in a study of their type of treatment as determined by their physician. The monitoring was done over a year with Moroccan Arabic dialect versions EORTC QLQ-C30 and EORTC QLQ-BR23 validated and standardized questionnaires.

Regarding EORTC QLQ-C30 questionnaire, our study showed that global health status has improved after a year of monitoring. This observation is on line with other studies [10–12] which revealed a good global health status in patients with breast cancer similar to or better than that of a healthy population. This will probably result in relatively rapid normalization of health after breast cancer treatment.

All scales of functional dimension (physical, role, emotional and cognitive) of EORTC QLQ-C30 showed high scores that have improved over time except social functioning where there was a trend towards improvement. This could be explained by the fact that the disease has a significant incentive effect of change in social and family life of our patients. Our results are consistent with those of authors [11, 12] who reported a significant improvement in the quality of life of these different scales during follow-up. However, our results for social functioning appear to be inconsistent with those of authors [13].

As for the symptom scales of EORTC QLQ-C30, they revealed a significant decrease in the severity of symptoms for fatigue, pain, nausea and vomiting, insomnia and loss of appetite.

The attenuation of these conditions would probably be associated with the conduct of the therapeutic process. There was a tendency to decrease in symptom severity for dyspnea and constipation scales. However, there was a worsening of symptoms for diarrhea scale.

Our results differ from those of David V. et al. [11] who reported an improvement in all symptom scales and Kristin H et al. [12] where worsening dyspnea and diarrhea have been noted. It have been also noted a deterioration in financial situation of patients during the first year of follow-up. This would be due to the fact that most women have a lower social status and also do not have social security either. These same results have been reported by authors [11].

Analysis of functional dimensions of the EORTC QLQ-BR23 revealed a significant improvement in quality of life on the scales of body image and future perspective. These results are consistent with those of authors [11]. However, authors [14] reported deterioration in the quality of life for body image and future perspective after a year of follow-up. Sexual function and sexual enjoyment that had high scores have worsened during follow-up. This likely reflected the influence of many physical, psychological and somatic factors, especially in the case of young women [15, 16]. Authors [11] found cases of deterioration in the quality of life for sexual function and no significant change in sexual enjoyment.

Regarding the size of the symptoms of EORTC QLQ-BR23, there was a significant improvement in symptoms in the arms and breasts during follow-up and worsening of symptoms on treatment side effects and hair loss during our study period. Authors [11] found a significant improvement in all symptoms of EORTC QLQ-C30 during follow-up.

## Conclusion

After this study, we could demonstrate a significant overall improvement in the quality of life of patients with breast cancer after a year of follow up regarding functional scales and symptom scales of the EORTC QLQ-C30 questionnaire. For the specific EORTC QLQ- BR23 questionnaire of breast cancer, there was observed a deterioration of the quality of life concerning sexual function and sexual enjoyment for functional scale and systemic therapy and hair loss for symptom scales. This study has shown that the evaluation of the quality of life in cancer patients could help improve treatment and also might even be a prognostic factor.

## Abbreviations

BC: Breast cancer
EORTC: European Organization for research and treatment of cancer
Qol: Quality of Life
